# Contrast-enhanced CT radiomics features to preoperatively identify differences between tumor and proximal tumor-adjacent and tumor-distant tissues of resectable esophageal squamous cell carcinoma

**DOI:** 10.1186/s40644-024-00656-0

**Published:** 2024-01-19

**Authors:** Dan Gao, Bang-guo Tan, Xiao-qian Chen, Chuanqinyuan Zhou, Jing Ou, Wen-wen Guo, Hai-ying Zhou, Rui Li, Xiao-ming Zhang, Tian-wu Chen

**Affiliations:** 1 Medical Imaging Key Laboratory of Sichuan Province, and Department of Radiology, Affiliated Hospital of North Sichuan Medical College, 1# Maoyuan South Road, Shunqing District, Nanchong, 637000 Sichuan China; 2 Department of Radiology, Medical Center Hospital of Qionglai City, 172# Xinglin Road, Linqiong District, Chengdu, 611530 Sichuan China; 3 Department of Radiology, Panzhihua Central Hospital, 34# Yikang Street, East District, Panzhihua, 617067 Sichuan China; 4 Department of Radiology, The Second Affiliated Hospital of Chongqing Medical University, 74# Linjiang Rd, Yuzhong District, Chongqing, 400010 China

**Keywords:** Esophagus, Squamous cell carcinoma, Tomography, X-ray computed, Tumor-adjacent tissue, Tumor-distant tissue

## Abstract

**Background:**

Esophagectomy is the main treatment for esophageal squamous cell carcinoma (ESCC), and patients with histopathologically negative margins still have a relatively higher recurrence rate. Contrast-enhanced CT (CECT) radiomics might noninvasively obtain potential information about the internal heterogeneity of ESCC and its adjacent tissues. This study aimed to develop CECT radiomics models to preoperatively identify the differences between tumor and proximal tumor-adjacent and tumor-distant tissues in ESCC to potentially reduce tumor recurrence.

**Methods:**

A total of 529 consecutive patients with ESCC from Centers A (*n* = 447) and B (*n* = 82) undergoing preoperative CECT were retrospectively enrolled in this study. Radiomics features of the tumor, proximal tumor-adjacent (PTA) and proximal tumor-distant (PTD) tissues were individually extracted by delineating the corresponding region of interest (ROI) on CECT and applying the 3D-Slicer radiomics module. Patients with pairwise tissues (ESCC vs. PTA, ESCC vs. PTD, and PTA vs. PTD) from Center A were randomly assigned to the training cohort (TC, *n* = 313) and internal validation cohort (IVC, *n* = 134). Univariate analysis and the least absolute shrinkage and selection operator were used to select the core radiomics features, and logistic regression was performed to develop radiomics models to differentiate individual pairwise tissues in TC, validated in IVC and the external validation cohort (EVC) from Center B. Diagnostic performance was assessed using area under the receiver operating characteristics curve (AUC) and accuracy.

**Results:**

With the chosen 20, 19 and 5 core radiomics features in TC, 3 individual radiomics models were developed, which exhibited excellent ability to differentiate the tumor from PTA tissue (AUC: 0.965; accuracy: 0.965), the tumor from PTD tissue (AUC: 0.991; accuracy: 0.958), and PTA from PTD tissue (AUC: 0.870; accuracy: 0.848), respectively. In IVC and EVC, the models also showed good performance in differentiating the tumor from PTA tissue (AUCs: 0.956 and 0.962; accuracy: 0.956 and 0.937), the tumor from PTD tissue (AUCs: 0.990 and 0.974; accuracy: 0.952 and 0.970), and PTA from PTD tissue (AUCs: 0.806 and 0.786; accuracy: 0.760 and 0.786), respectively.

**Conclusion:**

CECT radiomics models could differentiate the tumor from PTA tissue, the tumor from PTD tissue, and PTA from PTD tissue in ESCC.

## Background

Esophageal cancer is one of the most common malignant tumors threatening human health, and its mortality and morbidity rank 6th and 7th in the world, respectively [1]. Esophageal squamous cell carcinoma (ESCC) and adenocarcinoma are the main histological types, and ESCC accounts for approximately 90% of esophageal cancer cases worldwide [2, 3]. The overall 5-year survival rate of ESCC ranges from 15%—25% [4]. Currently, esophagectomy remains the mainstay treatment for ESCC [5], but in some patients with histopathologically negative margins, there is still a relatively higher recurrence rate. Previous studies revealed that field cancerization (FC) is a crucial factor in tumor recurrence [6, 7]. The notion of FC is that molecular changes in histologically normal adjacent tissues are similar to the tumor itself, which could lead to new invasive carcinomas in the resection margins or in “macroscopically normal” tissues adjacent to the tumor [7–10]. In addition, some previous studies reported FC approximately 1 cm away from the tumor margins (tumor-adjacent tissues) and near the ideal resection margins (tumor-distant tissues) in esophageal cancer patients, and found differences in gene expression and molecular mechanisms between tumor-adjacent and tumor-distant tissues [11, 12]. If the tissue showing precancerous lesions is not completely removed by surgery, it may cause local recurrence or develop into a second primary tumor [13]. In addition, transthoracic esophagotomy plus proximal partial gastrectomy were the main surgical methods for ESCC, and esophagogastric anastomosis was used to restore the continuity of the digestive tract. Therefore, it is of vital importance to preoperatively identify differences between the tumor, proximal tumor-adjacent (PTA) and proximal tumor-distant (PTD) tissues to formulate optimal surgical strategies to reduce tumor recurrence.

Contrast-enhanced computed tomography (CECT) is the preferred imaging method for the preoperative diagnosis of ESCC and treatment decision making. However, CT is mainly used to evaluate the external morphological characteristics of tumors, rather than assessing intratumor heterogeneity. Radiomics can quantitatively analyze a large number of features extracted from traditional medical images, such as CT, and noninvasively provide potential information about the biological characteristics and internal heterogeneity of tumors [14, 15]. In recent years, radiomics has been widely used to noninvasively evaluate the preoperative staging, chemoradiotherapy response and postoperative recurrence of ESCC [16–18]. To the best of our knowledge, no study has assessed the differences between the tumor, PTA and PTD tissues of ESCC using CECT radiomics. Therefore, the purpose of this study was to develop and validate radiomics models based on CECT to preoperatively differentiate between the tumor, PTA and PTD tissues of ESCC.

## Methods

### Patients

The institutional ethics committee of our institution approved this retrospective study, and written informed consent was waived.

**Fig. 1:**
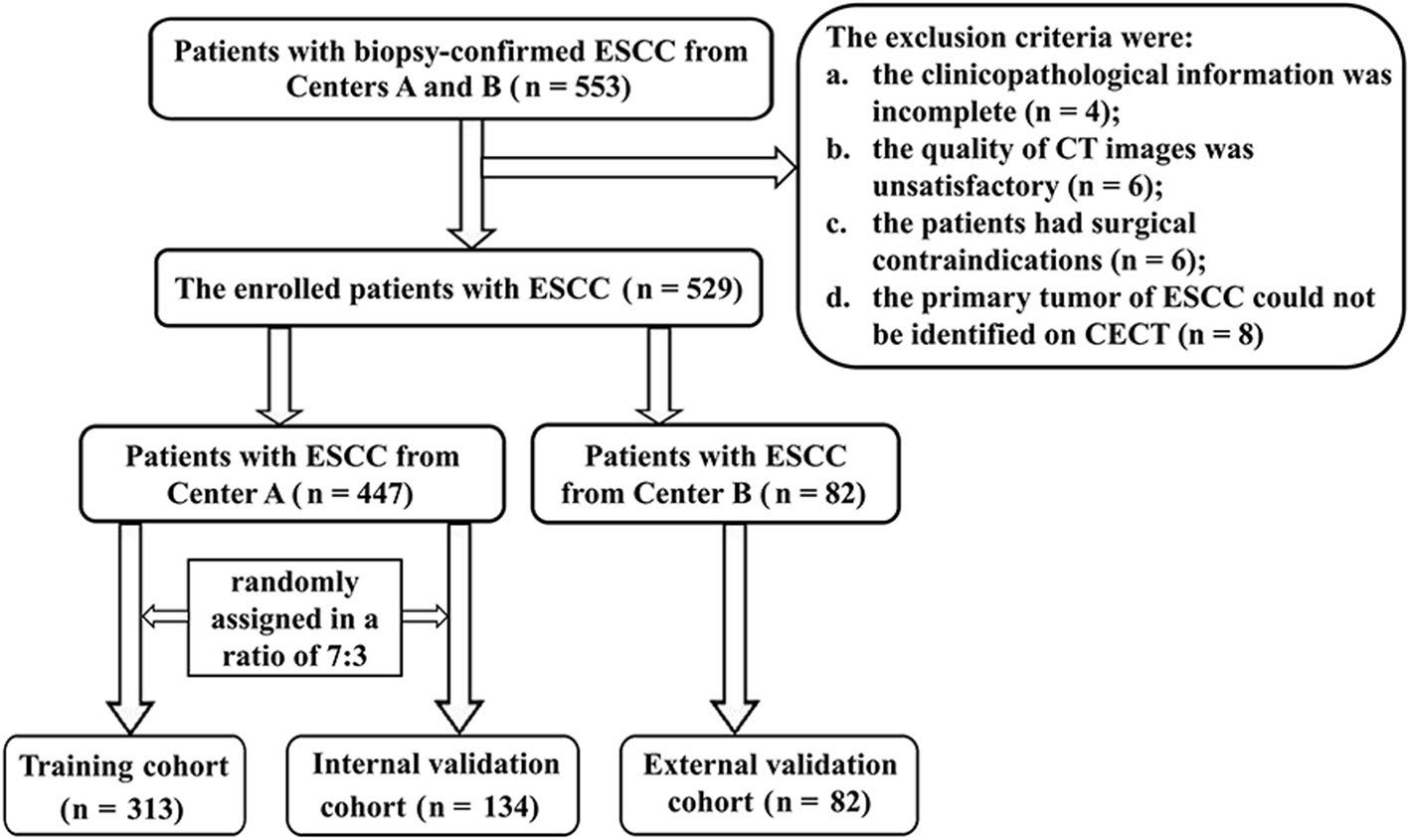
Flowchart for selecting the study population. Notes: CECT, contrast-enhanced computed tomography; ESCC, esophageal squamous cell carcinoma

From July 2020 to June 2022, we collected CT data and medical records of 553 consecutive patients with ESCC from Centers A and B. Patients were recruited into this study based on the following inclusion criteria: (a) patients underwent preoperative thoracic CECT scans within 2 weeks before surgery, and did not receive preoperative neoadjuvant chemotherapy and/or radiotherapy before undergoing CT examination; (b) ESCC was confirmed by preoperative endoscopy and pathological biopsy, and the surgical margin was postoperatively confirmed to be negative; (c) the patients had no history of other organ malignancies; and (d) the upper margin of the tumor was at least 5 cm away from the cricopharyngeal muscle. The exclusion criteria were as follows: (a) the clinicopathological information was incomplete (*n* = 4); (b) the quality of CT images was unsatisfactory (*n* = 6); (c) the patients had surgical contraindications (*n* = 6); or (d) the primary tumor of ESCC could not be identified on CECT (*n* = 8). Finally, 24 of 553 patients were excluded, and a total of 529 patients were involved in our study. Among the 529 patients, 447 patients from Center A were randomly assigned to the training cohort (TC, *n* = 313) and the internal validation cohort (IVC, *n* = 134) at a 7:3 ratio based on the published report [19], and 82 patients from Center B were assigned to the external validation cohort (EVC). The patient flowchart is displayed in Fig. 1.

All enrolled patients with ESCC received radical esophagectomy and regional lymph node dissection. The surgical margins in all resected specimens were not affected by the tumor (R0). Baseline clinical data of all patients, including age, sex, anatomical site of the primary tumor, differentiation degree, cT stage and cN stage, were obtained from the medical records (Table 1). Additionally, the differentiation degree, cT stage and cN stage were determined based on postoperative histopathological biopsy and the eighth edition American Joint Committee on Cancer guidelines [20]. In addition, patients with advanced ESCC determined by postoperative pathology received adjuvant treatments such as radiotherapy or chemotherapy.

**Table 1:** The clinical characteristics of patients from the Centers A and B

Parameters	Center A (*n* = 447)	Center B (*n* = 82)
Median age (years; range)	67 (42–85)	66 (63–84)
Gender (male: female)	332:115	58:24
Anatomic distribution (%)		
Upper thoracic portion	49 (11.0)	12 (14.6)
Middle thoracic portion	285 (63.7)	47 (57.3)
Lower thoracic portion	113 (25.3)	23 (28.1)
Differentiation (%)		
Well	169 (37.8)	30 (36.6)
Moderate	227 (50.8)	43 (52.4)
Poor	51 (11.4)	9 (11.0)
T stage (%)		
cT_1_	82 (18.4)	14 (17.1)
cT_2_	123 (27.5)	25 (30.5)
cT_3_	207 (46.3)	36 (43.9)
cT_4a_	35 (7.8)	7 (8.5)
N stage (%)		
cN_0_	263 (58.9)	41 (50.0)
cN_1_	128 (28.6)	24 (29.3)
cN_2_	56 (12.5)	17 (20.7)
Survival data		
Postoperative therapy (%)		
Yes	108 (24.2)	20 (24.4)
No	339 (75.8)	62 (75.6)
Disease-free survival within one year, months (mean ± SD)	11.22 ± 2.39	10.32 ± 3.67
Recurrence or death (%)		
Yes	100 (22.4)	22 (26.8)
No	347 (77.6)	60 (73.2)

*SD* standard deviation

### Follow-up

We followed up all enrolled patients every 3 to 6 months for one year or more after surgery through barium swallow, thoracoabdominal CT imaging or endoscopic biopsy, focusing on suspicious recurrence or the corresponding clinical symptoms related to postoperative recurrence, such as dysphagia. In addition, patients with confirmed recurrence after surgery underwent adjuvant treatments (such as radiotherapy or chemotherapy) to prolong their survival period. In each participant, the termination event of the follow-up was postoperative death during the follow-up period after surgery. The related survival data of all patients are also presented in Table 1.

### Image acquisition

All patients enrolled in our study underwent thoracic CECT scans with two 64 multidetector scanners (LightSpeed VCT, GE Medical systems, USA; and SOMATOM Definition AS + , Siemens Healthineers, Erlangen, Germany). Before CT data acquisition, 100 to 200 mL of water was needed to be drunk as oral esophageal negative contrast material. CT examinations were executed during one breath hold at full suspended inspiration for 10–15 s in the supine position. After a conventional unenhanced CT scan, CECT data acquisition was started 25 to 30 s after the initiation of 1.5 mL/kg nonionic iodine contrast agent (containing 300 mL/kg iodine) at a rate of 3.0 mL/s for a total of 70 to 100 mL via a 20-G needle inserted into an antecubital vein with a high-pressure injector and flushed with 20 mL saline. The parameters of the two CT scanners were as follows: tube voltage of 120 kV, tube current of 200 mA, detector collimation of 64 × 0.6 mm, reconstruction slice thickness of 1 mm, matrix of 512 × 512 mm for each scanner, rotation time of 0.5 s and 0.3 s, and pitch of 0.9 and 0.8 for the corresponding scanner. The anatomic coverage of the CT scan was from the chest entrance to the middle level of the left kidney. All the CECT data used for radiomics feature extraction were retrieved from the picture archiving and communication system.

### Image segmentation and radiomics feature extraction

To accurately extract the radiomics features of ESCC, PTA and PTD tissues, the definitions of the previous three tissues on CECT imaging were as follows. For ESCC, the esophageal wall thickness exceeding 5 mm was considered abnormal due to the primary tumor on axial imaging [21]. Clinically, the ideal proximal resection margin for ESCC is ≥ 5 cm [11, 22], so this study only investigated the characteristics of the proximal resection margin of ESCC. At a distance of 1 cm away from the tumor proximal margin, the molecular variation related to cancer is obvious, and this variation can also extend to the resection margin [11]. Therefore, we defined PTA and PTD as the tissues approximately 1 cm and 5 cm away from the proximal margin of the tumor, respectively.

**Fig. 2:**
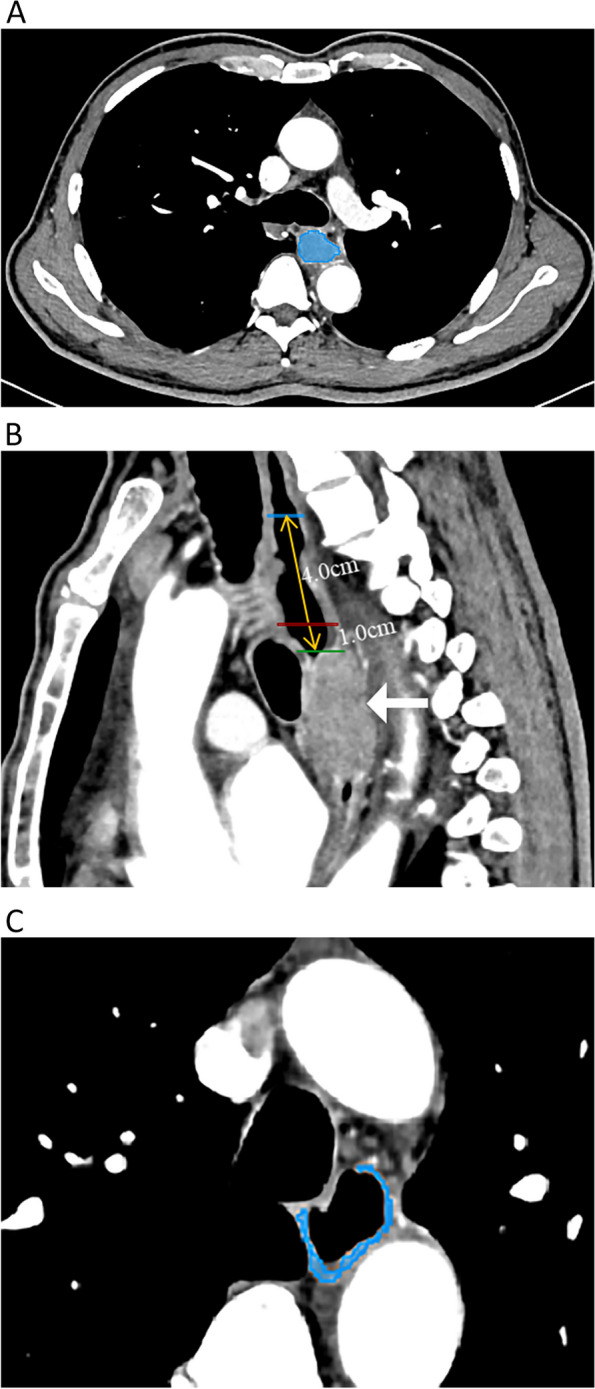
In a 64-year-old male patient with esophageal squamous cell carcinoma (ESCC), the region of interest (ROI) was delineated slice by slice on axial contrast-enhanced computed tomography (CECT) images (**A**). The white arrow refers to the primary tumor of ESCC; the blue, red and green solid lines represent the position lines of the proximal tumor-distant (PTD) tissue, proximal tumor-adjacent (PTA) tissue and the proximal margin of ESCC on the reconstructed sagittal CECT image, respectively; and the yellow arrow along the longitudinal axis of the esophagus indicates the ruler measuring line for defining the boundary lines of the PTD and PTA tissues (**B**). The ROI covering more than half of the esophageal wall for PTA tissue is manually drawn on an axial magnified CECT image (**C**). The ROI delineation of PTD tissue is performed similarly to that of PTA tissue

The thoracic CECT images of ESCC with 1 mm thickness were imported into 3D-Slicer (https://www.slicer.org), a free and open-source software, which was used for delineating regions of interest (ROIs) of the tumor (Fig. 2A), PTA and PTD tissues, and extracting radiomics features of the corresponding tissues. The locations of the PTA and PTD tissues were shown on the reconstructed sagittal CECT (Fig. 2B), and the axial ROIs of the PTA and PTD tissues were obtained at the corresponding distance (Fig. 2C). Two radiologists (readers 1 and 2, with 4 and 25 years of experience in digestive radiology, respectively) who were blinded to the patients’ pathological outcomes separately delineated ROIs of ESCC slice by slice and the ROIs of the PTA and PTD tissues (the area of each ROI more than half of the esophageal wall) on the magnified axial CECT scans, avoiding fat, air, bone and blood vessels. When two readers disagreed with each other, they achieved consensus after discussion and consultation. Finally, 1223 individual radiomics features of ESCC, PTA and PTD tissues were automatically extracted by applying the 3D-Slicer radiomics module, and radiomics features included first-order, gray-level co-occurrence matrix (GLCM), gray-level dependence matrix (GLDM), gray-level run-length matrix (GLRLM), gray-level size zone matrix (GLSZM), neighboring gray tone difference matrix (NGTDM), and shape.

### Intra- and interobserver agreements

To assess the repeatability of radiomics feature extraction, we randomly chose 60 consecutive samples from Center A to test the intra- and interobserver consistency by the previous 2 radiologists. The intraclass correlation coefficient (ICC) was used to evaluate the intra- and interobserver agreements. An ICC score greater than 0.75 indicated satisfactory agreement. To assess the intraobserver reproducibility, reader 1 sketched the ROIs of the tumor, PTA and PTD tissues twice within one week following the same delineating steps based on a published report [19]. In addition, reader 2 independently delineated the ROIs of the tumor, PTA and PTD tissues to assess the interobserver agreement by comparing the extracted radiomics features from the first ROI depiction by reader 1.

### Dimensionality reduction and radiomics feature selection

To avoid the curse of dimensionality and reduce the bias from radiomics features when modeling [19], we adopted the following three steps to select the significant features of the pairwise tissues (ESCC vs. PTA, ESCC vs. PTD, and PTA vs. PTD).

First, all the previous 1223 radiomics features underwent z-score normalization [23]: ${x}_{norm}=\frac{x-\mu }{\sigma }$, where *x* is the original feature value, *µ* is the mean value of this feature, and *σ* is the standard deviation.

Second, all features were examined by Student’s *t* test or the Mann‒Whitney U test to select potentially significant features from TC. Radiomics features that did not satisfy either of the aforementioned tests (*P* > 0.05) were excluded.

Third, the least absolute shrinkage and selection operator (LASSO) was used to identify the core radiomics features in TC to determine the differences between the previous pairwise tissues by performing variable selection and regularization of high-dimensional data, thus enhancing the accuracy and interpretability of the core radiomics features [19, 24]. The 1-standard error of the minimum criteria (the 1-SE criteria, a simpler model) was applied to adjust the regularization parameter (λ) for feature selection using 10-fold cross-validation.

### Construction and validation of the radiomics model

The optimal selected radiomics features from TC were used to build radiomics models based on logistic regression, a classical machine learning method. The radiomics models were used to evaluate the diagnostic performance of core radiomics features in TC for differentiating ESCC from PTA or PTD tissue and PTA from PTD tissue. The corresponding radiomics models of IVC and EVC were also obtained through the aforementioned logical regression method to validate the performance of the above radiomics models from TC. The area under the receiver operating characteristic curve (AUC), accuracy, F-1 score, sensitivity, specificity, positive predictive value (PPV), and negative predictive value (NPV) were calculated by the confusion matrix to evaluate the radiomics models based on the previous individual pairwise tissues of TC, IVC and EVC.

### Statistical analysis

All statistical analyses of radiomics data were conducted using R statistical software (version 4.2.1, https://www.r-project.org/). The “psych” package was used to evaluate the intra- and interobserver agreements of all radiomics features extracted from the corresponding ROIs of previous pairwise tissues of ESCC. LASSO regression based on multivariate logistic regression was performed using the “glmnet” and “pROC” packages, and the “pROC” package was used to plot the receiver operating characteristic (ROC) curves of TC, IVC and EVC. A *P* value less than 0.05 suggested a significant difference.

### Radiomics quality score

The readers 1 and 2 assessed the methodologic quality of our radiomics study in consensus through the radiomics quality score (RQS) proposed by Lambin in 2017 [25]. The RQS comprises 6 domains with 16 components. Domain 1, the assessment of the quality and replicability of image and segmentation; domain 2, the reporting of the feature reduction; domain 3, model performance and validation; domain 4, biological validation and potential clinical utility; and domains 5 and 6, demonstration of high-level evidence and open science, respectively [26]. Most items are designated to 0, 1 or 2 points. In order to highlight the importance of some dimensions, a higher point is assigned. For example, a prospective validation study is assigned 7 points, and a study validated in three or more datasets is assigned 5 points, while a study without validation is assigned -5 points. The ideal score of the RQS is 36 points, corresponding to a percentage of 100%.

## Results

### Intra- and interobserver agreements of feature extraction

**Fig. 3:**
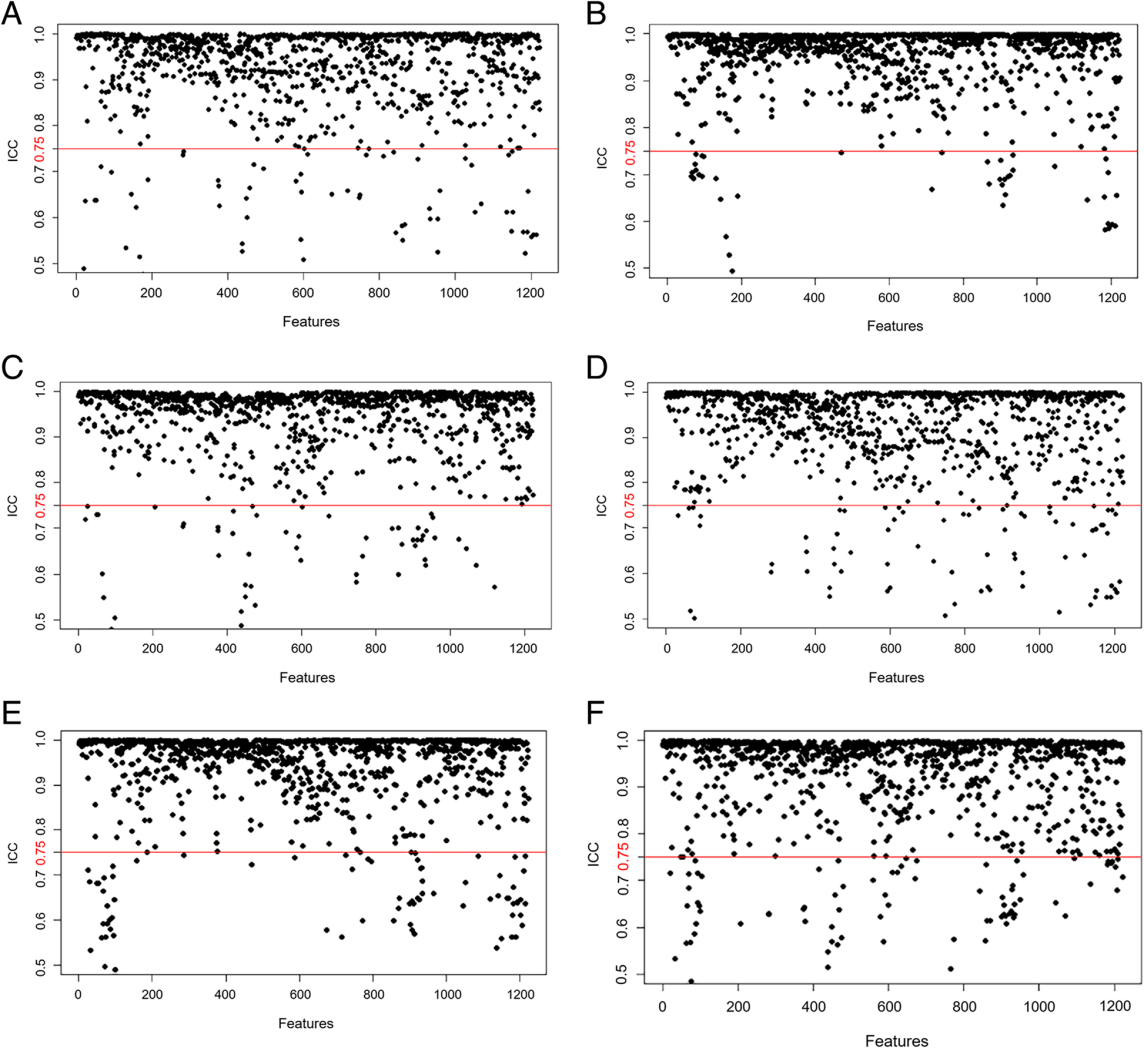
Feature stability evaluation with intra- and interobserver agreements based on the intraclass correlation coefficient (ICC). All features show good intraobserver agreement (**A**, **B** and **C**) and interobserver agreement (**D**, **E** and **F**) with ICCs > 0.75 (above the red cutoff line) in pairwise comparisons, including esophageal squamous cell carcinoma vs. proximal tumor-adjacent (PTA) tissue, tumor vs. proximal tumor-distant (PTD) tissue, and PTA vs. PTD tissue, respectively

For intra- and interobserver agreements of CECT radiomics feature extraction from the 60 random consecutive samples, there were 1122, 1137 and 1126 extracted features of the pairwise tissues (tumor vs. PTA, tumor vs. PTD, and PTA vs. PTD) with ICC values greater than 0.75, whereas there were 101, 86 and 97 unsatisfactory features with ICC values less than or equal to 0.75, respectively (Fig. 3). After this assessment, the 1122, 1137 and 1126 radiomics features with all intra- and interobserver ICC values of more than 0.75 were selected for further analyses. Reader 1 performed the segmentation and feature extraction of the remaining participants.

### Dimensionality reduction and radiomics feature selection

**Fig. 4:**
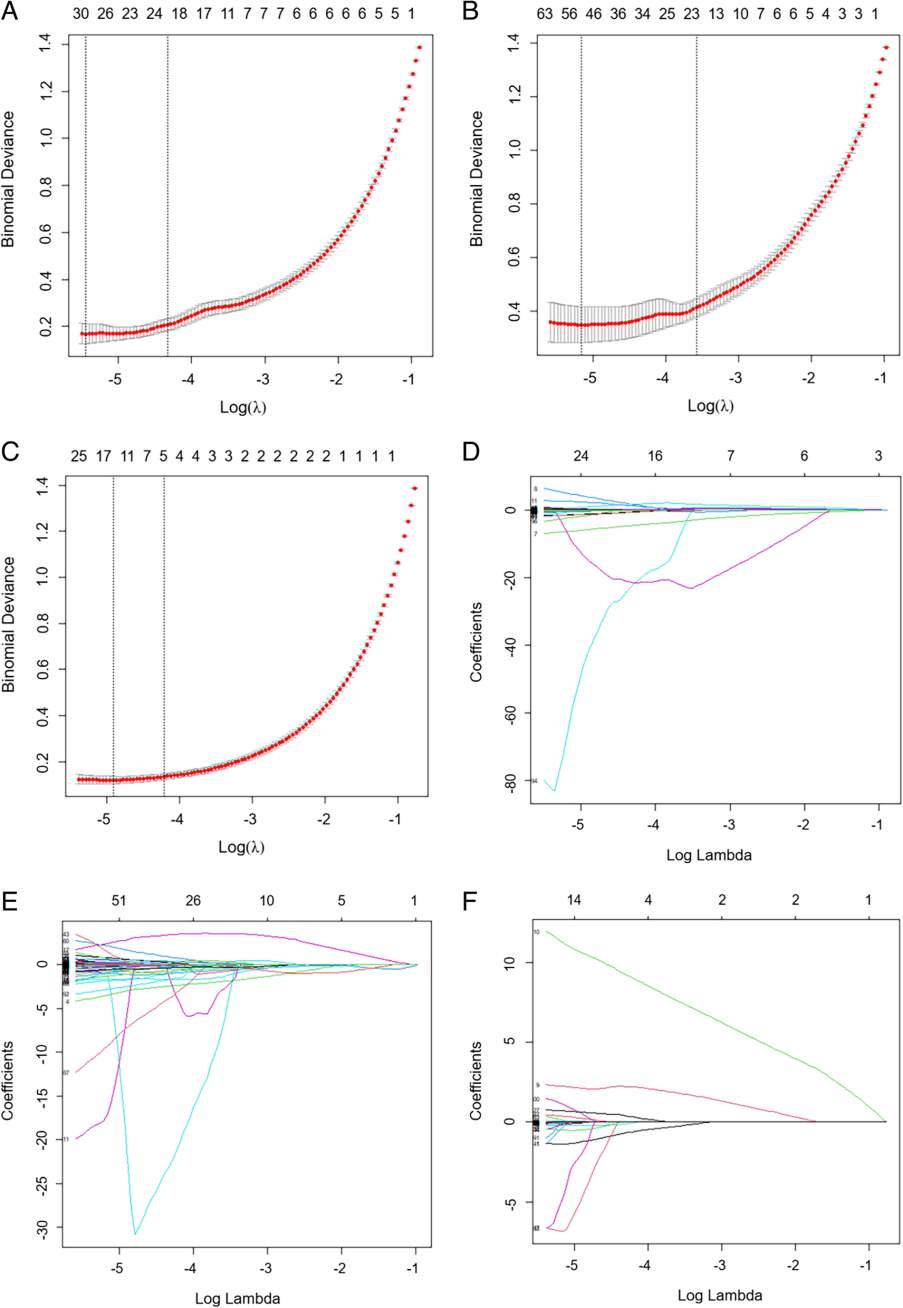
Radiomics feature selection using least absolute shrinkage and selection operator (LASSO) regression in pairwise tissues. The area under the receiver operating characteristic curve (AUC) was plotted by tuning the optimal parameter (λ) selection via 10-fold cross-validation and minimum criteria in the LASSO model. The left and right dotted lines denote the minimum criteria and the 1-standard error criterion (1-SE) in the pairwise tissues, including esophageal squamous cell carcinoma vs. proximal tumor-adjacent (PTA) tissue, the tumor vs. proximal tumor-distant (PTD) tissue, and the PTA vs. PTD tissue (**A**, **B** and **C**, respectively). The LASSO coefficient profiles the 1013, 1050 and 823 radiomics features for the differentiation of previous pairwise tissues, and as a result, 20 19 and 5 nonzero coefficients have been produced (**D**, **E** and **F**, respectively)

Among the previous 1122, 1137 and 1126 features in the pairwise tissues, including tumor vs. PTA, tumor vs. PTD, and PTA vs. PTD in all ESCC patients, the Student *t* test or Mann‒Whitney U test showed that 1013, 1050 and 823 features were statistically significant, respectively (all *P* values < 0.05). These features with statistical significance by the previous tests were separately used for the subsequent LASSO regression. For the pairwise tissues, including tumor vs. PTA, tumor vs. PTD, and PTA vs. PTD, 20, 19 and 5 features were screened (Tables 2, 3 and 4) to perform the corresponding differentiation, respectively, and 10-fold cross-validation was applied to select the best tuning regularization parameter (λ) under the 1-SE criteria (Fig. 4).

**Table 2:** The 20 features to differentiate tumor from proximal tumor-adjacent tissue of esophageal squamous cell carcinoma

Image type	Feature class	Feature name
Original	Shape	Elongation
Original	Shape	Maximum2DDiameterRow
Original	Shape	Maximum2DDiameterSlice
Original	Shape	SurfaceVolumeRatio
Log-sigma-1-0-mm-3D	Firstorder	Skewness
Log-sigma-1-0-mm-3D	Glcm	ClusterShade
Log-sigma-1-0-mm-3D	Glcm	Idmn
Log-sigma-1-0-mm-3D	Glcm	Idn
Log-sigma-1-5-mm-3D	Glrlm	ShortRunLowGrayLevelEmphasis
Log-sigma-1-5-mm-3D	Ngtdm	Coarseness
Log-sigma-2-0-mm-3D	Firstorder	90Percentile
Wavelet-LLH	Glcm	Correlation
Wavelet-LLH	Glam	LargeDependenceHighGrayLevelEmphasis
Wavelet-LLH	Gldm	SmallDependenceLowGrayLevelEmphasis
Wavelet-HHL	Glcm	Imc1
Wavelet-HHH	Glcm	MCC
Wavelet-HHH	Ngtdm	Coarseness
Wavelet-LLL	Firstorder	Median
Wavelet-LLL	Glcm	Correlation
Wavelet-LLL	Ngtdm	Coarseness

*Glcm* gray-level co-occurrence matrix, *Glrlm* gray-level run-length matrix, *Ngtdm* neighboring gray tone difference matrix, *Gldm* gray-level dependence matrix, *Idmn* inverse difference moment normalized, *Idn* inverse difference normalized, *Imc1* informational measure of correlation 1, *MCC* maximal correlation coefficient

**Table 3:** The 19 features to differentiate tumor from proximal tumor-distant tissue of esophageal squamous cell carcinoma

Image type	Feature class	Feature name
Original	Shape	Flatness
Original	Shape	Sphericity
Original	Shape	SurfaceVolumeRatio
Original	Firstorder	Median
Log-sigma-0-5-mm-3D	Glcm	Idmn
Log-sigma-0-5-mm-3D	Glszm	GrayLevelNonUniformity
Log-sigma-1-0-mm-3D	Firstorder	Kurtosis
Log-sigma-1-0-mm-3D	Glcm	Idmn
Log-sigma-2-0-mm-3D	Gldm	LargeDependenceEmphasis
Log-sigma-2-0-mm-3D	Glrlm	GrayLevelNonUniformity
Wavelet-LLH	Glcm	ClusterProminence
Wavelet-LLH	Glcm	MCC
Wavelet-LLH	Glrlm	ShortRunHighGrayLevelEmphasis
Wavelet-LHL	Glcm	Imc2
Wavelet-HLL	Glcm	Imc1
Wavelet-HLL	Glcm	Imc2
Wavelet-HLL	Ngtdm	Strength
Wavelet-HHH	Glszm	SmallAreaEmphasis
Wavelet-LLL	Glcm	Correlation

*Glcm* gray-level co-occurrence matrix, *Glszm* gray-level size zone matrix, *Gldm* gray-level dependence matrix, *Glrlm* gray-level run-length matrix, *Ngtdm* neighboring gray tone difference matrix, *Idmn* inverse difference moment normalized, *MCC* maximal correlation coefficient, *Imc1* informational measure of correlation 1, *Imc2* informational measure of correlation 2

**Table 4:** The 5 features to differentiate PTA from PTD of esophageal squamous cell carcinoma

Image type	Feature class	Feature name
Original	Shape	Elongation
Original	Shape	Maximum2DDiameterColumn
Original	Shape	Maximum2DDiameterRow
Log-sigma-0-5-mm-3D	Firstorder	Median
Wavelet-HLH	Glcm	Imc1

*PTA* proximal tumor-adjacent tissue, *PTD* proximal tumor-distant tissue, *Glcm* gray-level co-occurrence matrix, *Imc1* informational measure of correlation 1

### Construction and validation of the radiomics models

**Fig. 5:**
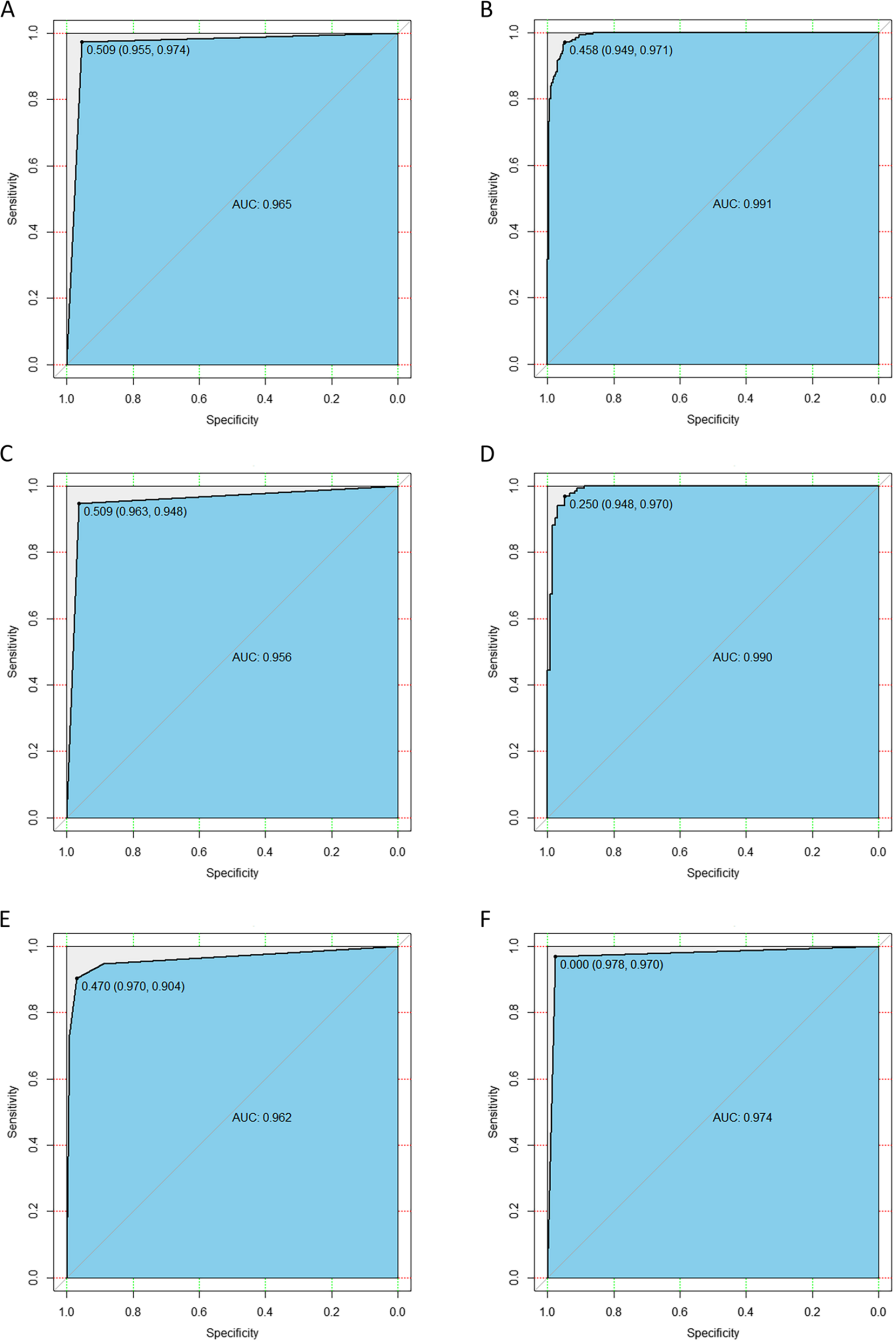
The receiver operating characteristic (ROC) curves of the radiomics models for differentiating between esophageal squamous cell carcinoma and proximal tumor-adjacent tissue and between the tumor and proximal tumor-distant tissue in the training cohort (**A** and **B**, respectively), internal validation cohort (**C** and **D**, respectively), and external validation cohort (**E** and **F**, respectively)

**Fig. 6:**
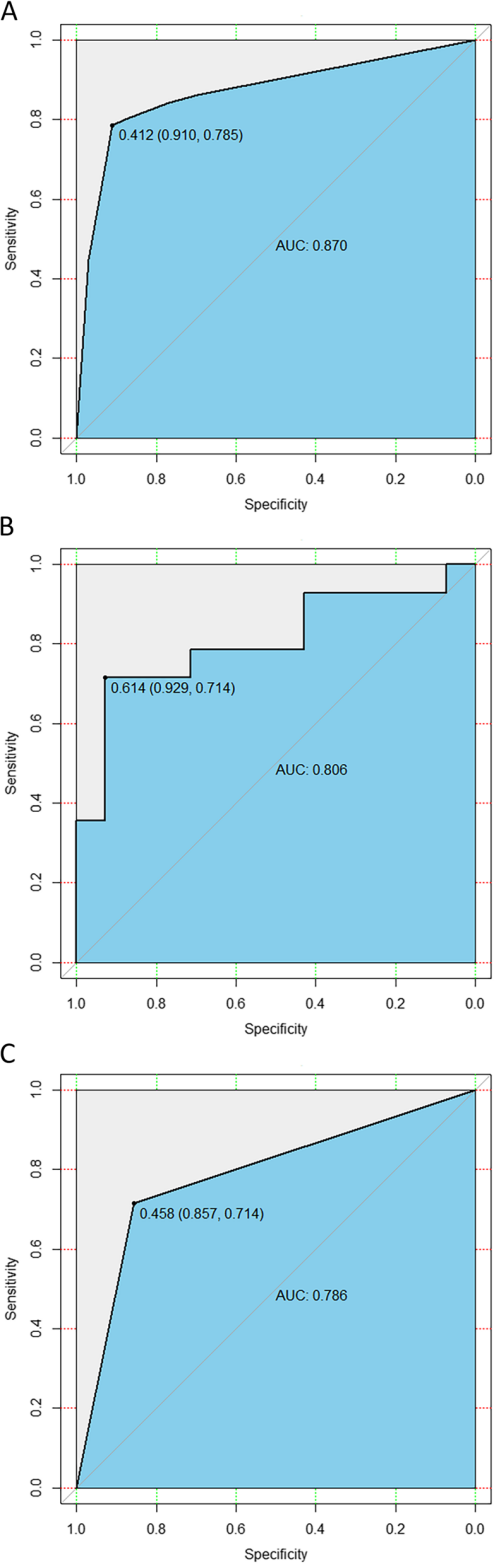
The receiver operating characteristic (ROC) curves of the radiomics model for differentiating between proximal tumor-adjacent tissue and tumor-distant tissue in the training cohort (**A**), internal validation cohort (**B**) and external validation cohort (**C**)

Through logistic regression, the above 20, 19 and 5 selected core radiomics features were applied to build individual radiomics models for identifying the differences between the tumor and PTA tissue, the tumor and PTD tissue, and the PTA and PTD tissues in TC, respectively. Then, the 3 models for the individual pairwise tissues were validated by the IVC and EVC. The most appropriate individual models selected by AUC, accuracy, F-1 score, sensitivity, specificity, PPV, and NPV are illustrated in Table 5. The ROC curves (Fig. 5) visually revealed that the individual radiomics models had excellent ability to differentiate the tumor from PTA tissue and the tumor from PTD tissue in TC, and effective performance for the previous differential diagnoses in the IVC and EVC. As shown in Fig. 6, the logistic radiomics model showed good performance for differentiating the PTA from PTD tissue in TC and was also helpful for distinguishing the PTA from PTD tissue in the IVC and EVC.

**Table 5:** Performance of the radiomics models to differentiate between tumor, PTA and PTD of ESCC

Pairwise tissues	Cohort	AUC (95%CI)	Accuracy	F1-score	Sensitivity	Specificity	PPV	NPV
Tumor vs. PTA	TC	0.965 (0.950-0.979)	0.965	0.965	0.974	0.955	0.956	0.974
	IVC	0.956 (0.931-0.980)	0.956	0.955	0.948	0.963	0.962	0.949
	EVC	0.962 (0.939-0.985)	0.937	0.935	0.904	0.970	0.968	0.910
Tumor vs. PTD	TC	0.991 (0.985-0.997)	0.958	0.959	0.971	0.949	0.953	0.964
	IVC	0.990 (0.979-1.000)	0.952	0.951	0.970	0.948	0.946	0.969
	EVC	0.974 (0.955-0.993)	0.970	0.969	0.970	0.978	0.977	0.963
PTA vs. PTD	TC	0.870 (0.841-0.898)	0.848	0.838	0.785	0.910	0.897	0.809
	IVC	0.806 (0.632-0.980)	0.760	0.759	0.714	0.929	0.865	0.769
	EVC	0.786 (0.630-0.941)	0.786	0.769	0.714	0.857	0.833	0.750

*ESCC* esophageal squamous cell carcinoma, *PTA* proximal tumor-adjacent tissue, *PTD* proximal tumor-distant tissue, *TC* training cohort, *IVC* internal validation cohort, *EVC* external validation cohort, *AUC* area under the receiver operating characteristic curve, *95%CI* 95% confidence interval, *PPV* positive predictive value, *NPV* negative predictive value

### The RQS of our radiomics study

According to the RQS evaluation criteria and reporting guidelines, the RQS score of our study was 16, accounting for 44.4% of the total points. The scores for each item are listed in Table 6.

**Table 6:** The assessment of the radiomics quality score

No	RQS component (score range)	Points
1	Image protocol quality (0 to 2)	1
2	Multiple segmentation (0 to 1)	1
3	Phantom study on all scanners (0 to 1)	0
4	Imaging at multiple time points (0 to 1)	0
5	Feature reduction or adjustment for multiple testing (-3 to 3)	3
6	Multivariable analysis with non-radiomic features (0 to 1)	0
7	Detect and discuss biological correlates (0 to 1)	0
8	Cut-off analyses (0 to 1)	0
9	Discrimination statistics (0 to 2)	2
10	Calibration statistics (0 to 2)	0
11	Prospective study registered in a trial database (0 to 7)	0
12	Validation (-5 to 5)	4
13	Comparison to gold standard (0 to 2)	2
14	Potential clinical utility (0 to 2)	2
15	Cost-effectiveness analysis (0 to 1)	0
16	Open science and data (0 to 4)	1

## Discussion

In the current study, we initially developed and validated individual CECT radiomics models for the preoperative differentiation of tumor from PTA tissue, tumor from PTD tissue, and the PTA from PTD tissue in ESCC.

As shown in this study, 1122 and 1137 candidate radiomics features with ICCs > 0.75 in the pairwise tissues (including ESCC vs. PTA and ESCC vs. PTD) were reduced to 20 and 19 core features to develop the individual radiomics models to effectively differentiate the tumor from PTA tissue and the tumor from PTD tissue of ESCC, and the previous core features were composed of 13 and 14 texture features, 3 and 2 first-order features, and 4 and 3 shape features, respectively. Some selected features, such as skewness in the first-order feature class, appear to be related to the differentiation of the tumor from PTA or PTD tissue; however, it is challenging to reliably link a single radiomics feature with the complex pathological state of different tissues [27]. Therefore, constructing and validating multifeature models is a more feasible and reliable approach to potentially differentiate the tumor from PTA tissue and tumor from PTD tissue. Our CECT radiomics models showed excellent diagnostic performance in TC, IVC and EVC, with all AUCs of more than 0.95, indicating that our radiomics models could play a pivotal role in differentiating the tumor from PTA tissue and the tumor from PTD tissue.

Moreover, we also selected 5 core features from the 1126 features with ICC > 0.75 in the pairwise tissue (PTA vs. PTD) to develop the CT radiomics model to differentiate between the PTA and PTD tissues of ESCC. The 5 core features include 1 texture feature, 1 first-order feature and 3 shape features. The texture feature and the first-order feature most likely reflect the differences in microarchitecture and internal heterogeneity between the PTA and PTD tissues. Shape features exhibit the external contour information of the PTA and PTD tissues. Our study revealed that the radiomics model developed with the previous 5 core features could help differentiate the PTA from PTD tissue in TC (AUC: 0.870), as validated by IVC (AUC: 0.806) and EVC (AUC: 0.786). For the first time, our study developed a CT radiomics model for discriminating the PTA from PTD tissue of ESCC, and we can speculate that differentiation in radiomics features between the PTA and PTD tissues might contribute to preoperative evaluation of the extent of surgical resection to some degree.

In our research, the following methods are adopted to ensure robustness. First, to ensure the accuracy of radiomics feature extraction, we drew the ROI along the tumor edge based on a reference criterion that the thickness of the abnormal esophageal wall was more than 5 mm [28, 29], which has been proven reasonable. For PTA and PTD tissues, we depicted the area of each ROI in at least more than half of the esophageal wall to obtain sufficient CECT image information. Second, intra- and interobserver agreements, univariate analysis and LASSO were used for feature selection, thus guaranteeing the independence and accuracy of each feature extraction in the final models. We used 10-fold cross-validation and stepwise regression to avoid overfitting of the data and guarantee the robustness of the models [24]. Third, our study added EVC to validate the performance of the radiomics models in TC, which ensured the reliability and stability of the models for differentiating the tumor from PTA tissue, the tumor from PTD tissue, and the PTA from PTD tissue. Fourth, RQS was used to assess the methodologic quality of our radiomics research, and our study obtained a score of 16, greater than or equal to the scores of several relevant articles in the published meta-analysis review [30], indicating our current radiomics models could help guide the design of our future radiomics study to improve the reliability and repeatability of radiomics models for clinical application.

There are still several limitations in our study. First, our study is a retrospective study. A prospective study will be needed in the future to further verify our results. Second, we depicted the ROIs of PTA and PTD tissues at only two levels (1 cm and 5 cm away from the proximal margin of the tumor) and did not investigate the differences in radiomics features of the surrounding-tumor tissues at different sections away from the proximal margin of the tumor. We will conduct relevant research in the future. Third, we did not consider the proteomic and metabolomic characteristics at present. P53 protein accumulation and metabolic profile generation in the tumor and proximal histologically normal tissue have been proven to be evidence for field cancerization in patients with esophageal cancer [12, 31]. We will combine radiomics with proteomics or metabolomics for our future studies. Last but not least, an existing study [32] has shown that cancer biology might evolve during neoadjuvant treatment, resulting not only in a chemoradiotherapy-resistant residue but also possibly a more aggressive cancer. In addition, we did not know whether neoadjuvant therapy would impact the radiomics features of the tumor or the PTA and PTD tissues of resectable ESCC in patients with advanced cancer. Therefore, our study only enrolled patients who did not undergo neoadjuvant therapy to ensure the accuracy and reliability of the extracted radiomics features of the tumor and the PTA and PTD tissues of resectable ESCC.

## Conclusions

Our study innovatively developed and validated individual CECT radiomics models using core radiomics features in pairwise tissues (including tumor vs. PTA, tumor vs. PTD, and PTA vs. PTD), and the radiomics models were proven to be valuable in differentiating the tumor from PTA tissue, the tumor from PTD tissue, and the PTA from PTD tissue. We hope that our radiomics models could be helpful for formulating surgical decision-making to reduce postoperative local recurrence.

## Data Availability

Please contact the corresponding author (Dr. Tian-wu Chen) for data requests.

## Abbreviations

ESCC: Esophageal squamous cell carcinoma
FC: Field cancerization
PTA: Proximal tumor-adjacent
PTD: Proximal tumor-distant
CECT: Contrast-enhanced computed tomography
TC: Training cohort
IVC: Internal validation cohort
EVC: External validation cohort
ROI: Regions of interest
GLCM: Gray-level co-occurrence matrix
GLDM: Gray-level dependence matrix
GLRLM: Gray-level run-length matrix
GLSZM: Gray-level size zone matrix
NGTDM: Neighboring gray tone difference matrix
ICC: Intraclass correlation coefficient
LASSO: Least absolute shrinkage and selection operator
AUC: Area under the receiver operating characteristic curve
PPV: Positive predictive value
NPV: Negative predictive value
ROC: Receiver operating characteristic

## References

[1] Heymach J, Krilov L, Alberg A, Baxter N, Chang SM, Corcoran RB (2018). Clinical cancer advances 2018: annual report on progress against cancer from the American society of clinical oncology. J Clin Oncol.

[2] Wang SM, Abnet CC, Qiao YL (2019). What have we learned from Linxian esophageal cancer etiological studies?. Thorac Cancer.

[3] Rice TW, Gress DM, Patil DT, Hofstetter WL, Kelsen DP, Blackstone EH (2017). Cancer of the esophagus and esophagogastric junction-Major changes in the American Joint Committee on Cancer eighth edition cancer staging manual. CA Cancer J Clin.

[4] Pennathur A, Gibson MK, Jobe BA, Luketich JD (2013). Oesophageal carcinoma. Lancet.

[5] Takeuchi H, Miyata H, Gotoh M, Kitagawa Y, Baba H, Kimura W (2014). A risk model for esophagectomy using data of 5354 patients included in a Japanese nationwide web-based database. Ann Surg.

[6] Slaughter DP, Southwick HW, Smejkal W (1953). “Field cancerization” in oral stratified squamous epithelium: clinical implications of multicentric origin. Cancer.

[7] Tabor MP, Brakenhoff RH, Ruijter-Schippers HJ, Kummer JA, Leemans CR, Braakhuis BJM (2004). Genetically altered fields as origin of locally recurrent head and neck cancer: a retrospective study. Clin Cancer Res.

[8] Heaphy CM, Griffith JK, Bisoffi M (2009). Mammary field cancerization: molecular evidence and clinical importance. Breast Cancer Res Treat.

[9] Bugter O, Spaander MCW, Bruno MJ, Baatenburg de Jong RJ, Amelink A, Robinson DJ (2018). Optical detection of field cancerization in the buccal mucosa of patients with esophageal cancer. Clin Transl Gastroenterol.

[10] Chen XQ, Tan BG, Xu M, Zhou HY, Ou J, Zhang XM (2022). Apparent diffusion coefficient derived from diffusion-weighted imaging to differentiate between tumor, tumor-adjacent and tumor-distant tissues in resectable rectal adenocarcinoma. Eur J Radiol.

[11] Matsuda Y, Yamashita S, Lee YC, Niwa T, Yoshida T, Gyobu K (2012). Hypomethylation of Alu repetitive elements in esophageal mucosa, and its potential contribution to the epigenetic field for cancerization. Cancer Causes Control.

[12] Yakoub D, Keun HC, Goldin R, Hanna GB (2010). Metabolic profiling detects field effects in nondysplastic tissue from esophageal cancer patients. Cancer Res.

[13] Braakhuis BJM, Tabor MP, Leemans CR, van der Waal I, Snow GB, Brakenhoff RH (2002). Second primary tumors and field cancerization in oral and oropharyngeal cancer: molecular techniques provide new insights and definitions. Head Neck.

[14] Gillies RJ, Kinahan PE, Hricak H (2016). Radiomics: images are more than pictures, they are data. Radiology.

[15] Lambin P, Rios-Velazquez E, Leijenaar R, Carvalho S, Granton P, Gillies R (2012). Radiomics: extracting more information from medical images using advanced feature analysis. Eur J Cancer.

[16] Wu L, Wang C, Tan X, Cheng ZX, Zhao K, Yan LF (2018). Radiomics approach forpreoperative identification of stages I-II and III-IV of esophageal cancer. Chin J Cancer Res.

[17] Hou Z, Ren W, Li S, Liu J, Sun Y, Yan J (2017). Radiomic analysis in contrast-enhanced CT: predict treatment response to chemoradiotherapy in esophageal carcinoma. Oncotarget.

[18] Tang S, Jing OU, Liu J, Wu YP, Wu CQ, Chen TW (2021). Application of contrast-enhanced CT radiomics in prediction of early recurrence of locally advanced oesophageal squamous cell carcinoma after trimodal therapy. Cancer Imaging.

[19] Chen Y, Chen TW, Wu CQ, Lin Q, Hu R, Xie CL (2019). Radiomics model of contrast-enhanced computed tomography for predicting the recurrence of acute pancreatitis. Eur Radiol.

[20] Rice TW, Ishwaran H, Ferguson MK, Blackstone EH, Goldstraw P (2017). Cancer of the esophagus and esophagogastric junction: an eighth edition staging primer. J Thorac Oncol.

[21] Moss AA, Schnyder P, Thoeni RF, Margulis AR (1981). Esophageal carcinoma: pretherapy staging by computed tomography. AJR Am J Roentgenol.

[22] Müller JM, Erasmi H, Stelzner M, Zieren U, Pichlmaier H (1990). Surgical therapy of oesophageal carcinoma. Br J Surg.

[23] Cheadle C, Vawter MP, Freed WJ, Becker KG (2003). Analysis of microarray data using Z score transformation. J Mol Diagn.

[24] Shafiq-Ul-Hassan M, Zhang GG, Latifi K, Ullah G, Hunt DC, Balagurunathan Y (2017). Intrinsic dependencies of CT radiomic features on voxel size and number of gray levels. Med Phys.

[25] Lambin P, Leijenaar RTH, Deist TM, Peerlings J, de Jong EEC, Timmeren J (2017). Radiomics: the bridge between medical imaging and personalized medicine. Nat Rev Clin Oncol.

[26] Zhou HY, Cheng JM, Chen TW, Zhang XM, Ou J, Cao JM (2023). CT radiomics for prediction of microvascular invasion in hepatocellular carcinoma: a systematic review and meta-analysis. Clinics (Sao Paulo).

[27] Jiang Y, Chen C, Xie J, Wang W, Zha XF, Lv WB (2018). Radiomics signature of computed tomography imaging for prediction of survival and chemotherapeutic benefits in gastric cancer. EBioMedicine.

[28] Dionigi G, Rovera F, Boni L, Bellani M, Bacuzzi A, Carrafiello G (2006). Cancer of the esophagus: the value of preoperative patient assessment. Expert Rev Anticancer Ther.

[29] Tan XZ, Ma ZL, Yan LF, Ye WT, Liu ZY, Liang CH (2019). Radiomics nomogram outperforms size criteria in discriminating lymph node metastasis in resectable esophageal squamous cell carcinoma. Eur Radiol.

[30] Kao YS, Hsu Y (2021). A meta-analysis for using radiomics to predict complete pathological response in esophageal cancer patients receiving neoadjuvant chemoradiation. In Vivo.

[31] Tian D, Feng Z, Hanley NM, Setzer RW, Mumford JL, DeMarini DM (1998). Multifocal accumulation of p53 protein in esophageal carcinoma: evidence for field cancerization. Int J Cancer.

[32] de Gouw DJJM, Klarenbeek BR, Driessen M, Bouwense SAW, van Workum F, Fütterer JJ (2019). Detecting pathological complete response in esophageal cancer after neoadjuvant therapy based on imaging techniques: a diagnostic systematic review and meta-analysis. J Thorac Oncol.

